# Significant difference between three observers in the assessment of intraepidermal nerve fiber density in skin biopsy

**DOI:** 10.1186/1471-2377-9-13

**Published:** 2009-03-31

**Authors:** Sigrid Wöpking, Andrea Scherens, Ida S Haußleiter, Helmut Richter, Julia Schüning, Sabrina Klauenberg, Christoph Maier

**Affiliations:** 1 Department of Pain Management, Ruhr-University Bochum, BG-Kliniken Bergmannsheil, Bürkle-de-la-Camp-Platz 1, D-44789 Bochum, Germany; 2 Department of Neurology, Ruhr-University Bochum, BG-Kliniken Bergmannsheil, Bürkle-de-la-Camp-Platz 1, D-44789 Bochum, Germany

## Abstract

**Background:**

The determination of Intraepidermal Nerve Fiber Density (IENFD) in skin biopsy is a useful method for the evaluation of different types of peripheral neuropathies. To allow a reliable use of the method it is necessary to determine interobserver reliability. Previous studies dealing with this topic used limited suitable statistical methods.

**Methods:**

In the present study three observers determined the IENFD and estimated the staining quality of the basement membrane for an adequate quantity of 120 skin biopsies (stained with indirect immunofluorescence technique) from 68 patients. More adequate statistical methods like intraclass correlation coefficient and Bland Altman Plot were chosen to estimate interobserver reliability.

**Results:**

We found an unexpected significant difference in IENFD between the observers (p < 0.05) and so the results of this study are not in line with the high interobserver reliability reported before (intraclass correlation coefficient: 0.73). The Bland Altmann Plot showed a variance growing with rising mean. The difference in IENFD between the observers and the resulting low interobserver reliability is likely caused by different interpretations of the standard counting rules. There was no significant difference in IENFD between observers for biopsies with a well-defined basement membrane. Thus skin biopsies with an inexactly defined basement membrane should not be used diagnostically for the determination of IENFD.

**Conclusion:**

These results emphasise that standardisation of the method is extremely important and at least two observers should analyse skin biopsies with critical IENFD near the cut-off values.

## Background

Despite the fact that numerous patients in pain or neurology departments are admitted for typical neuropathic symptoms such as paraesthesia and dysaesthesia the conventional diagnostic methods such as nerve conduction studies and electromyography often do not show pathological findings [1-4]. Immunohistochemical illustration of the intraepidermal nerve fibers (IENF) in skin biopsy and quantitative sensory testing (QST) are two new diagnostic methods to objectify the disorders of some of these patients [5]. In 2005 Lauria et al. published the guidelines of the European Federation of Neurological Societies (EFNS) on the use of skin biopsy and the determination of IENF density (IENFD) in the diagnosis of peripheral neuropathy [6]. For a reliable use of this method, a check of methodical quality criteria is essential. Especially reliability as a degree of methodical accuracy has to be determined, e.g. by calculating the interobserver reliability. Therefore two or more observers conduct the same test and their accordance is subsequently analysed.

A few previous studies deal with interobserver reliability [7-10]. Some of them by calculating the correlation coefficients [7,10]. The value of such correlation coefficients to determine interobserver reliability is limited, since level differences remain unnoticed and extreme values can pretend a higher reliability [11]. Smith et al. calculated the intraclass correlation coefficient and the relative intertrial variability (RIV) to determine interobserver reliability. Special calculations apply for the RIV ([(IENFD_1_-IENFD_2_)/MW _(IENFD)_] *100 [%]) and values less than 10% indicate a high degree of reproducibility. Small absolute differences at low IENFD values are presented as high percentage values while equivalent absolute differences at high IENFD values are being presented as lower percentage values [9]. This approach can lead to an incorrect estimation of the reliability. Gøransson et al. estimated the interobserver reliability by calculating the absolute difference between the IENFD results of two observers.

Due to the limited suitability of the statistical methods so far applied, there is still some need to adequately demonstrate interobserver reliability of the IENFD determination by skin biopsy. To achieve this, three independent observers analysed a sufficient quantity of biopsies. Additionally, more appropriate statistical methods were chosen in order to confirm a reliable use of the skin biopsy in clinical diagnostics.

## Methods

### 2.1 Patients

Skin biopsies from 68 patients were examined, who all previously participated in several independent studies designed to examine the validity of QST and to determine the IENFD in skin biopsy. Patients suffered from polyneuropathy (n = 23), fibromyalgia (n = 18), arthritis (n = 13) or neuropathic pain after nerve injury at the lower limb (n = 14). The duration of symptoms of patients with nerve injury ranged from 14 to 294 months, with a median of 46 months. Affected nerves were either the common or superficial peroneal nerve (n = 11) or the lateral cutaneous nerve of the thigh (n = 3). The age of all patients ranged from 21 to 74 years (mean 52 ± 13 years) (Table 1). All studies were approved by the local ethics committee of the Ruhr University Bochum and the patients gave written informed consent.

**Table 1:** Demographic characteristics of patients with polyneuropathy, nerve injury at the lower limb, fibromyalgia and arthritis

Diagnosis	Polyneuropathy	Nerve injury	Fibromyalgia	Arthritis	**Total**
Number of subjects (n)	23	14	18	13	**68**
Age (year), Mean ± SD	58.96 ± 10.62	41 ± 13.29	49.06 ± 10.25	57.23 ± 10.19	52.31 ± 13.07
Age (year), Range	36–74	21–67	27–68	26–68	21–74
Sex, Male (n)	13	11	4	3	31

### 2.2 Skin biopsy

The procedure of skin biopsy followed the protocol by Vlckova-Moravcova et al. [12], as a modified version of the original Guidelines of the EFNS [6]. Indirect immunofluorescence technique was used. Two samples were taken from each patient, one from the affected and one from an unaffected skin area. In patients with polyneuropathy, fibromyalgia and arthritis biopsies were therefore carried out from dorso-lateral foot and back (dermatome L4). The very distal biopsy site at the foot was chosen because all patients had complaints at this area, but not all had complaints at the lower leg, which would be the standard biopsy site recommended by the EFNS guidelines. As a level A recommendation those guidelines also suggest the sampling of an additional biopsy from an unaffected site in patients with generalised diseases to provide information about a length-dependent process. L4 dermatome was assessed as a second area, which was the least affected area in most of the patients. In patients with nerve injury biopsies were carried out bilaterally from foot (dorsolateral or dorsomedial) or lateral thigh. After local injection of 2% lidocaine the removal was carried out under sterile conditions with a 3 mm biopsy punch (Stiefel GmbH, Offenbach, Germany). Tissue was fixed in 4% phosphate-buffered paraformaldehyde for 3–4 hours and cryoprotected in 10% sucrose at 4°C overnight. Subsequently the skin samples were embedded in TissueTek^®^, frozen in 2-methylbutane cooled in liquid nitrogen and stored at 70°C until further processing. Sections of 40 μm thickness were cut on a sliding microtome and immunostained with rabbit polyclonal antibodies to human PGP 9.5 (Ultraclone, UK, 1:800) as primary antibody and marked with Cyanine 3 (Jackson Immuno Research, USA). The intraepidermal nerve fibers were counted manually in two sections of approximately 3 mm length each by three independent observers (MF, ISH, SW), who were professionally trained at an approved skin biopsy laboratory (Department of Neurology, University of Würzburg, Germany). Counting was conducted in a blinded fashion to determine interobserver reliability at 400× magnification with a Zeiss Axiophot 2 microscope adhering to standard counting rules [13], agreed on by the European guidelines 2005 [6]. Samples were only evaluated if the staining quality of both sections were judged to be satisfactory by all observers (e.g. distinct discrimination of dermis and epidermis, clearly illustrated nerve fibers). Samples were excluded for the determination of interobserver reliability if they were judged to be of bad quality for counting by at least one observer (e.g. nerve fibers or basement membrane stained badly). Using Image Pro Plus 4.0 software (Media Cybernetics, Leiden, The Netherlands), the epidermal length was accurately measured. The average intraepidermal nerve fiber density (IENFD) per mm of epidermal length was then calculated. IENFD results from biopsies taken from the foot were compared with published control data [12] as done in a previous study [4] and classified as pathologic in case of IENFD less than 9 fibers/mm.

Additionally every observer evaluated the definition of the basement membrane in each biopsy, classifying it as 'well', 'moderately' or 'inexactly' defined. In summary the basement membrane was rated 'well defined' if at least two observers ranked it so.

### 2.3 Data analysis

All statistical analyses were performed using the Statistica software package, release 7.1 for Windows (StatSoft Inc., USA) and the statistical package for social sciences (SPSS 12). Differences between observers were analysed using a one-way analysis of variance (ANOVA). Due to the unprovable homogeneity of variance post hoc comparisons were calculated using Dunnet T3 post hoc tests. P values < 0.05 were considered significant. Since IENFD from adjacent sections of one biopsy showed a high degree of association [7], the accuracy of each observer was estimated by calculating the standard deviation between both sections of one biopsy (intersection variability) and the relative standard deviation (SD/mean). To demonstrate the variance growing with rising mean the results were presented as Bland Altman Plot [14]. Interobserver reliability was measured by calculating intraclass correlation coefficient with absolute agreement definition [15]. To compare the results of this study with those of previous studies correlation coefficients and RIV were also measured. For the RIV applies ([(IENFD_1_-IENFD_2_)/MW _(IENFD)_] *100 [%]) and values of less than 10% indicate a high degree of reproducibility [9].

## Results

A total of 120 biopsies from 68 patients (polyneuropathy: n = 44; nerve injury at the lower limb: n = 25; fibromyalgia: n = 30; athritis: n = 21) were analysed. 16 biopsies had to be excluded due to bad quality.

**Figure 1:**
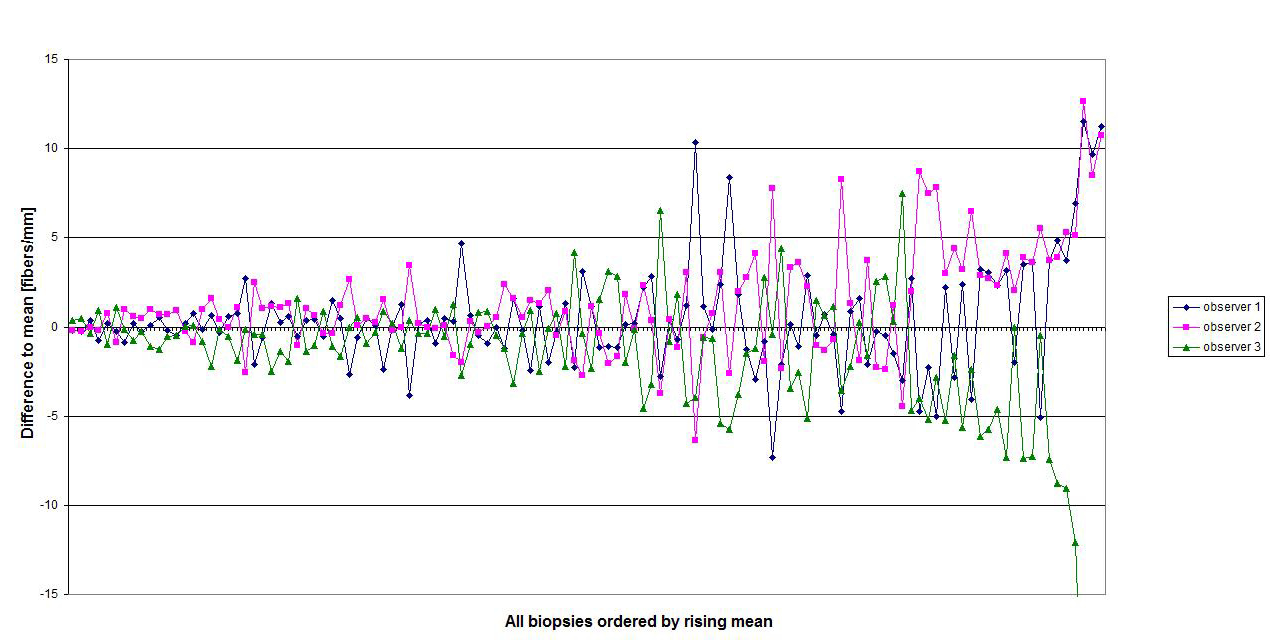
**Bland Altman Plot for all biopsies (n = 120)**.

Evaluation of the complete data showed a significant difference between the IENFD counted by different observers (Table 2). Variance increased with rising mean (Figure 1). However, even at low IENFD values, e.g. in biopsies taken from the foot, the difference between the observers remained significant. Overall, observer 2 counted the highest values for all biopsy sites, whereas observer 3 stated the lowest values for all biopsy sites. In conformity with these results the Post Hoc tests revealed that in all cases the significant difference laid only between these two observers.

**Table 2:** IENFD by different observers for skin biopsy sites foot, back and thigh and total data

Skin biopsy site	Observer 1	Observer 2	Observer 3	Significance
**Foot, n = 71**	**IENFD [fibers/mm]**	**IENFD [fibers/mm]**	**IENFD [fibers/mm]**	p

Mean ± SD	4.88 ± 4.1	5.16 ± 4.00	3.57 ± 2.56	*
Range	0–22.8	0–23.8	0–13.21	-
Median	3.90	4.04	3.02	-
25–75% percentile	2.17–6.55	2.66–6.61	1.93–4.59	-
Inter section SD	0.76	0.84	0.5	**
Relative inter section SD	0.21	0.21	0.17	-

**Back, n = 45**	**IENFD [fibers/mm]**	**IENFD [fibers/mm]**	**IENFD [fibers/mm]**	-

Mean ± SD	15.97 ± 11.63	17.74 ± 11.73	12.20 ± 4.78	*
Range	0–56.08	0.54–55.61	1.7–22.88	-
Median	11.92	16.21	11.95	-
25–75% percentile	7.78–21.8	7.32–24.44	9.06–15.18	-
Inter section SD	1.67	2.09	1.16	-
Relative inter section SD	0.1	0.15	0.11	-

**Thigh, n = 4**	**IENFD [fibers/mm]**	**IENFD [fibers/mm]**	**IENFD [fibers/mm]**	-

Mean ± SD	4.15 ± 2.49	5.69 ± 4.21	2.12 ± 2.68	-
Range	1.38–8.13	2.07–12.86	0.19–6.68	-

**Total, n = 120**	**IENFD [fibers/mm]**	**IENFD [fibers/mm]**	**IENFD [fibers/mm]**	-

Mean ± SD	9.01 ± 9.48	9.89 ± 9.93	6.76 ± 5.52	*
Range	0–56.08	0–55.61	0–22.88	-
Median	5.51	6.29	4.86	-
25–75% percentile	3.1–11.2	3.69–12.37	2.31–11.14	-
Inter section SD	1.11	1.31	0.74	*
Relative inter section SD	0.17	0.19	0.16	-

Significance: * p < 0.05 ** p < 0.01

The intersection variability differed significantly between the observers for the foot data and the complete data. Observer 3 had the lowest values in contrast to observer 2 who had the highest ones (table 2). In this case the Post Hoc tests revealed a significant difference between observer 3 and both other observers for the foot data. However, with respect to the overall data the intersection variability differed significantly between observer 2 and 3.

The comparison of IENFD results of 71 foot biopsies with published control data showed that the significant interobserver difference would generate different rates of pathological results. The results from observer 3 would add up to 68 pathological biopsies in opposition to the other observers with lower numbers of pathological biopsies (62 and 63 respectively). Since the control data were taken from the distal calf [12] the accuracy of the comparison results might be limited.

**Figure 2:**
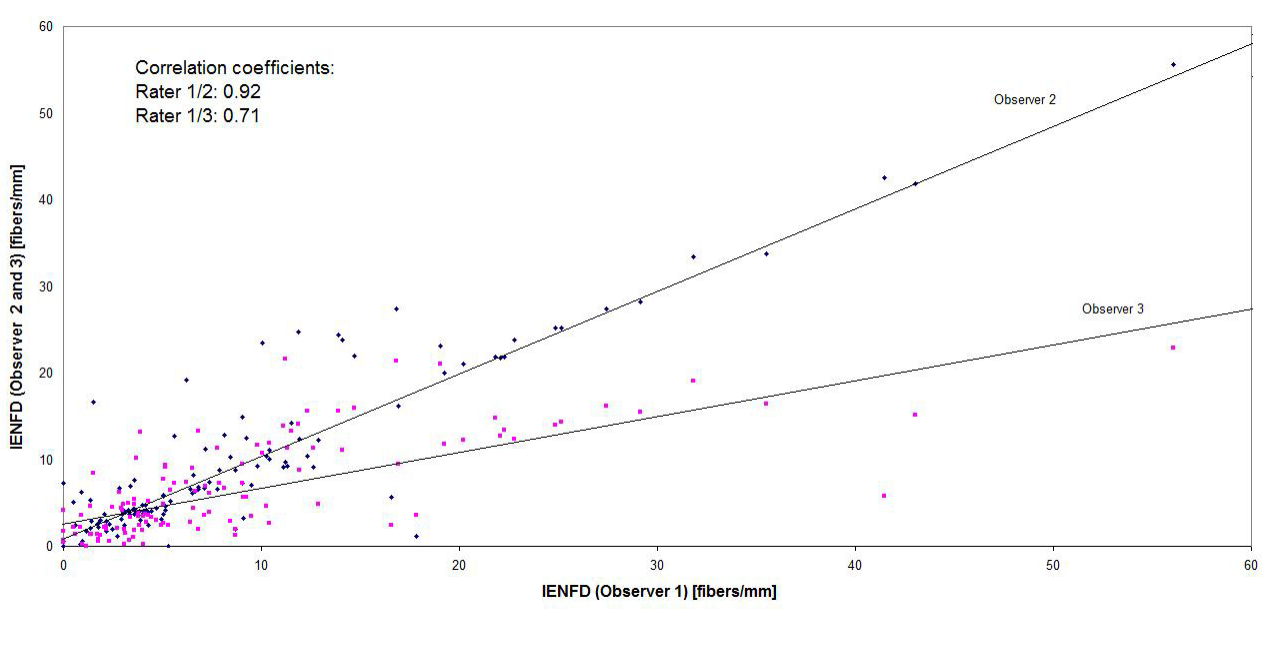
**Correlation between IENFD measured by three independent observers for all biopsies (n = 120) (Correlation coefficients for observer 1/2 and 1/3)**.

The intraclass correlation coefficient for all data was 0.73. Due to the significant difference between the observers the correlation coefficients (Figure 2 + 3) and RIV with participation of observer 3 showed the lowest values. The RIV was 35.6% between observer 1 and 2, 61% between observer 1 and 3 and 63.8% between observer 2 and 3.

**Figure 3:**
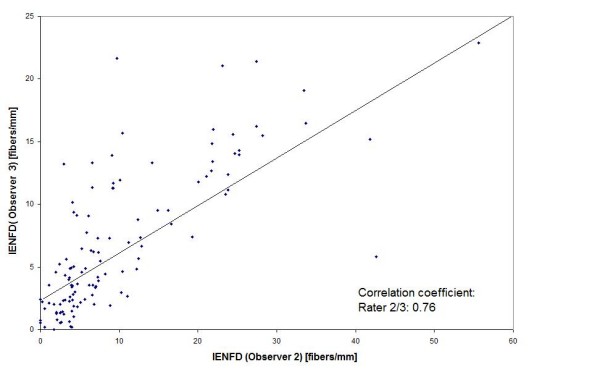
**Correlation between IENFD measured by three independent observers for all biopsies (n = 120)**. (Correlation coefficient for observer 2/3).

The basement membrane has been rated as "well defined"in 35 biopsies. Observer 3 also found lower values for these biopsies than the other observers, but the difference was not significant (Table 3). Correlation coefficients changed and especially those with observer 3 showed higher values (observer 1/2: r = 0,85; observer 1/3: r = 0,8; observer 2/3: r = 0,85). The intraclass correlation coefficient increased sligthly (0.77) as well.

**Table 3:** IENFD by different observers for punches with well defined dermal-epidermal basement membrane (n = 35).

	Observer 1	Observer 2	Observer 3
Mean ± SD	8.74 ± 7.72	9.82 ± 7.93	6.85 ± 5.2
Range	0–27.41	0–27.41	0.52–16.22
Median	7.12	7.04	5.02
25–75% percentile	2.17–12.32	3.66–14.21	2.31–11.95

No significant difference between observers

## Discussion

Our results revealed an unexpected significant difference in IENFD between three observers. Despite having received the same training, the three observers most likely interpreted the standard counting rules [13] differently.

**Figure 4:**
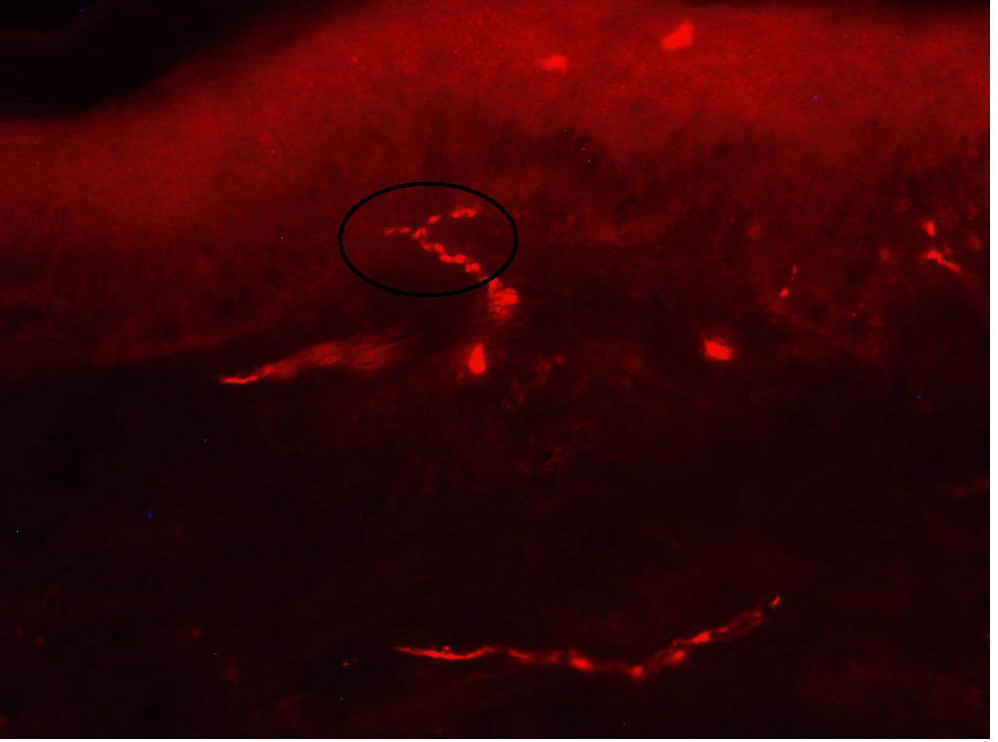
**Skin biopsy immunostained for PGP 9.5, which were evaluated in this study**. Number of IENF stated by the observers (MF, ISH and SW) of this study: 0–2 fibers. The different results were probably caused by difficulties to determine the correct position of the fiber: (e.g.: the fiber approaches the basement membrane but do not cross it → 0 fibers, the fiber branches after crossing the basement membrane → 1 fiber, the fiber branches within the basement membrane → 2 fibers.

**Figure 5:**
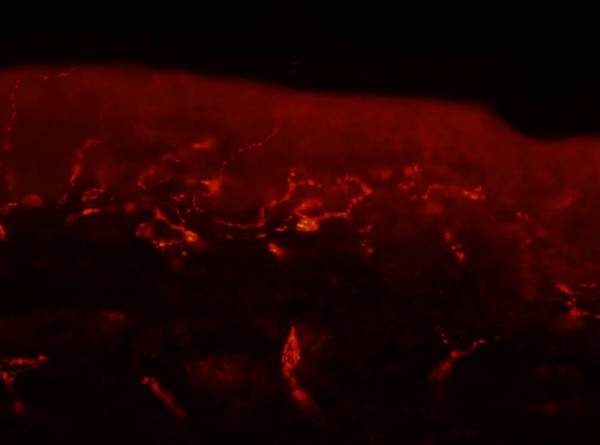
**Skin biopsy immunostained for PGP 9.5, which were evaluated in this study**. Number of IENF stated by the observers (MF, ISH and SW) of this study: 7–11 fibers. The different results were probably caused by difficulties to determine the correct position of the fibers due to the high number of fibers and inexact illustration of nerve fibers and basement membrane.

Due to a less clear and accurate staining of the skin innervation in microscopic sections an observer might have difficulties to count the intraepidermal nerve fibers exactly according to the counting rules. This problem occured in biopsies with low IENFD values (figure 4) as well as in biopsies with high IENFD values (figure 5). Some observers tended to a broader interpretation (Figure 4: 2 fibers, figure 5: 11 fibers) of the counting rules, whilst others interpreted them more strictly (Figure 4: 0 fibers, figure 5: 7 fibers). From a clinical point of view broader interpretation bears the risk of missing pathological biopsies (low sensitivity, high specifity), whereas stricter interpretation implicates the risk to rate healthy biopsies as pathological (low specifity, high sensitivity). The different frequencies of pathological values in the foot biopsies in this study underline that this might be a relevant problem in clinical diagnostics.

Since we found the lowest values of intersection variability and therefore the highest accuracy for the observer stating the lowest IENFD values, the strict interpretation might be more reliable.

Other groups stated higher interobserver reliability with correlation coefficients ranging from 0.86–0.96 [7,10]. In further studies the RIV was 9.6%, the intraclass correlation coefficient 0.98 [9] and the mean difference between the IENFD results of two observers 0.4 ± 1.5 fibers/mm [8]. The low interobserver reliability in our study was probably caused by the described significant interobserver difference in IENFD. Additionally we might have found higher interobserver reliability by counting three sections as recommended by the EFNS Guidelines [6]. Considering the pronounced significant difference between the observers in our study, the results would have probably been similar. Furthermore an accessory analysis of intra-observer reliability would allow a more accurate interpretation of the interobserver reliability.

The qualitative evaluation of the basement membrane before counting the intraepidermal nerve fibers could be an approach to improve the methodical accuracy. The results allow the conclusion that interobserver reliability is higher if the basement membrane is well defined. Consequently skin biopsies with inexact illustration of the basement membrane should not be used for the determination of IENFD in clinical diagnostics and scientific studies. However the number of biopsies with a well defined basement membrane was quite small in our study and there was only a little improvement of interobserver reliability.

Another possibility to avoid inaccurate IENF counting due to an inexactly defined basement membrane might be the use of antibodies against collagen IV with confocal microscopy to better visualise the basement membrane [6].

## Conclusion

In summary, the determination of IENFD by skin biopsy is a useful method to investigate different types of peripheral neuropathy [16], but our results show that standardisation of the method is extremely important. However the number of biopsies was quite small in our study and we used a modified version of the original Guidelines of the EFNS. Therefore our results are limited to a small number of patients but lead us to following conclusion. To avoid inaccurate IENFD counting, clear inclusion and exclusion criteria for skin biopsy samples should be further defined. The EFNS Guidelines [6] recommend the application of the counting protocol which was described by Kennedy et al [13]. Our results show that a consensus should be reached on the interpretation of the counting rules in biopsies with less accurate illustration of the skin innervation. We recommend that observers undergo thorough training and intraobserver reliability must be demonstrated by intra-lab assessment to avoid different interpretation of the counting rules by individuals. Nevertheless IENFD counting may still be a subjective investigation partially. Skin biopsies with critical IENFD values (IENFD near the cut-off values) should be analysed by at least two observers together. Furthermore, mandatory external quality controls of skin biopsy laboratories e.g. by interlaboratory comparison should be enforced. Whilst in experienced laboratories the interobserver reliability may not an issue, consensus data is still needed for application to all labs.

## Competing interests

The authors declare that they have no competing interests.

## Authors' contributions

SW participated in the design of the study, evaluated skin biopsies, conducted analysis and interpretation of data and participated to draft the manuscript. AS participated in the design of the study, made analysis and interpretation of data and participated to draft the manuscript. ISH participated in the design of the study, evaluated skin biopsies and participated to draft the manuscript. HR performed the statistical analysis. JS recruited the patients suffering from nerve injury and helped to draft the manuscript. SK recruited the patients suffering from fibromyalgia or arthritis. CM participated in the design of the study, made analysis and interpretation of data and participated to draft the manuscript. All authors read an approved the final manuscript.

## Pre-publication history

The pre-publication history for this paper can be accessed here:



## References

[B1] Holland NR, Crawford TO, Hauer P, Cornblath DR, Griffin JW, McArthur JC (1998). Small-fiber sensory neuropathies: clinical course and neuropathology of idiopathic cases. Ann Neurol.

[B2] Gibbons CH, Griffin JW, Polydefkis M, Bonyhay I, Brown A, Hauer PE, Mc Arthur JC (2006). The utility of skin biopsy for prediction of progression in suspected small fiber neuropathy. Neurology.

[B3] Fink E, Oaklander AL (2006). Small-fiber neuropathy: answering the burning questions. Sci Aging Knowledge Environ.

[B4] Scherens A, Maier C, Haussleiter IS, Schwenkreis P, Vlckova-Moravcova E, Baron R, Sommer C (2008). Painful or painless lower limb dysesthesias are highly predictive of peripheral neuropathy: comparison of different diagnostic modalities. Eur J Pain.

[B5] Løseth S, Lindal S, Stalberg E, Mellgren SI (2006). Intraepidermal nerve fibre density, quantitative sensory testing and nerve conduction studies in a patient material with symptoms and signs of sensory polyneuropathy. Eur J Neurol.

[B6] Lauria G, Cornblath DR, Johansson O, McArthur JC, Mellgren SI, Nolano M, Rosenberg N, Sommer C, European Federation of Neurological Societies (2005). EFNS guidelines on the use of skin biopsy in the diagnosis of peripheral neuropathy. Eur J Neurol.

[B7] McArthur JC, Stocks EA, Hauer P, Cornblath DR, Griffin JW (1998). Epidermal nerve fiber density: normative reference range and diagnostic efficiency. Arch Neurol.

[B8] Gøransson LG, Mellgren SI, Lindal S, Omdal R (2004). The effect of age and gender on epidermal nerve fiber density. Neurology.

[B9] Smith AG, Howard JR, Kroll JR, Ramachandran P, Hauer P, Singleton JR, McArthur J (2005). The reliability of skin biopsy with measurement of intraepidermal nerve fiber density. J Neurol Sci.

[B10] Koskinen M, Hietaharju A, Kyläniemi M, Peltola J, Rantala I, Udd B, Haapasalo H (2005). A quantitative method for the assessment of intraepidermal nerve fibers in small-fiber neuropathy. J Neurol.

[B11] Weiß C, Weiß C (2002). Die Korrelationsanalyse. Basiswissen Medizinische Statistik.

[B12] Vlcková-Moravcová E, Bednarik J, Dusek L, Toyka KV, Sommer C (2008). Diagnostic validity of epidermal nerve fiber densities in painful sensory neuropathies. Muscle Nerve.

[B13] Kennedy WR, Wendelschafter-Crabb G, Polydefkis M, Mc Arthur J, Dyck PJ, Thomas PK (2005). Pathology and quantitation of cutaneous nerves. Peripheral Neuropathy.

[B14] Bland JM, Altman DG (1986). Statistical methods for assessing agreement between two methods of clinical measurement. Lancet.

[B15] Shrout PE, Fleiss JL (1979). Intraclass Correlations: Uses in Assessing Rater Reliability. Psychological Bulletin.

[B16] Sommer C, Lauria G (2007). Skin biopsy in the management of peripheral neuropathy. Lancet Neurol.

